# Ca^2+^ spark latency and control of intrinsic Ca^2+^ release dyssynchrony in rat cardiac ventricular muscle cells

**DOI:** 10.1016/j.yjmcc.2023.07.005

**Published:** 2023-07-09

**Authors:** Cherrie H.T. Kong, Mark B. Cannell

**Affiliations:** School of Physiology, Pharmacology and Neuroscience, Faculty of Biomedical Sciences, University of Bristol, University Walk, Bristol BS8 1TD, United Kingdom

**Keywords:** Heart, Ca^2+^ sparks, Cardiac myocytes, Excitation-contraction coupling, Action potential, Intracellular Ca^2+^

## Abstract

Cardiac excitation-contraction coupling (ECC) depends on Ca^2+^ release from intracellular stores via ryanodine receptors (RyRs) triggered by L-type Ca^2+^ channels (LCCs). Uncertain numbers of RyRs and LCCs form ‘couplons’ whose activation produces Ca^2+^ sparks, which summate to form a cell-wide Ca^2+^ transient that switches on contraction. Voltage (V_m_) changes during the action potential (AP) and stochasticity in channel gating should create variability in Ca^2+^ spark timing, but Ca^2+^ transient wavefronts have remarkable uniformity. To examine how this is achieved, we measured the V_m_-dependence of evoked Ca^2+^ spark probability (P_spark_) and latency over a wide voltage range in rat ventricular cells. With depolarising steps, Ca^2+^ spark latency showed a U-shaped V_m_-dependence, while repolarising steps from 50 mV produced Ca^2+^ spark latencies that increased monotonically with V_m_. A computer model based on reported channel gating and geometry reproduced our experimental data and revealed a likely RyR:LCC stoichiometry of ~ 5:1 for the Ca^2+^ spark initiating complex (IC). Using the experimental AP waveform, the model revealed a high coupling fidelity (P_cpl_ ~ 0.5) between each LCC opening and IC activation. The presence of ~ 4 ICs per couplon reduced Ca^2+^ spark latency and increased P_spark_ to match experimental data. Variability in AP release timing is less than that seen with voltage steps because the AP overshoot and later repolarization decrease P_spark_ due to effects on LCC flux and LCC deactivation respectively. This work provides a framework for explaining the V_m_- and time-dependence of P_spark_, and indicates how ion channel dispersion in disease can contribute to dyssynchrony in Ca^2+^ release.

## 1 Introduction

Contraction of the heart depends critically on the near-synchronous Ca^2+^ release from internal stores (the sarcoplasmic reticulum, SR) via a process called ‘Ca^2+^-induced Ca^2+^ release’ (CICR) [1]. During normal excitation-contraction coupling (ECC), stochastic activation of surface membrane L-type Ca^2+^ channels (LCCs) supplies the Ca^2+^ trigger for CICR. Amplification of the Ca^2+^ influx via LCCs (i_Ca_) [2,3] is provided by the Ca^2+^-dependent gating of multiple ryanodine receptors (RyRs, [4,5]) in the SR membrane. RyRs are clustered in dyads where the SR membrane is closely opposed to the surface membrane [6,7]). The local Ca^2+^ changes arising from LCC and RyR gating in this dyadic space are limited to “couplons” [8] where spatially restricted Ca^2+^ release events (“Ca^2+^ sparks” [9]) eventuate. This “local control” [2,8] of CICR normally prevents uncontrolled cell-wide regenerative Ca^2+^ release and is the result of high local Ca^2+^ concentrations in the dyad being transduced by relatively insensitive RyRs (that are unlikely to open at cytoplasmic levels of Ca^2+^) (for review see [10]).

Current models for ECC have focussed on the general features of relevant ion channel gating (e.g. [11,12]), nanoscopic structure of the dyad (e.g. [13,14]) and clustering of RyRs (e.g. [15,16]) in ventricular cells to contribute to a coherent explanation for the probabilistic nature of Ca^2+^ spark production [8,11,17,18]. However, it is unclear whether our existing knowledge of these processes is sufficient to explain both the membrane voltage- (V_m_) and time-dependence of Ca^2+^ spark initiation (especially in response to an AP). The paucity of kinetic data on the timing of Ca^2+^ spark initiation is problematic for ECC models that should provide the biophysical underpinning for understanding both normal and abnormal Ca^2+^ release. Nevertheless, experiments have shown that the normal ventricular Ca^2+^ transient has remarkable uniformity [2,19] despite the intrinsic stochasticity in LCC and RyR gating that should cause temporally non-uniform Ca^2+^ release across dyads [2].

In the present study, we have analyzed the V_m_-dependence of evoked Ca^2+^ spark timing and probability and compared the experimental data to predictions of an integrative computer model for Ca^2+^ spark initiation. We show that simple step command voltage clamp protocols, as are usually employed in biophysical experiments, do not reproduce the physiological latency for AP evoked Ca^2+^ sparks. An explanation for this unexpected observation is provided by the computer model, which was able to reproduce the experimental data. The model also allows exploration of the likely RyR:LCC stoichiometry that is needed to produce Ca^2+^ release with a short latency, as well as how many LCC openings are needed to initiate Ca^2+^ sparks (called the “coupling fidelity” [20]).

## 2 Methods

### 2.1 Myocyte isolation and Ca^2+^ imaging

All procedures were carried out in accordance with the UK Home Office Animals (Scientific Procedures) Act 1986 and conformed to the guidelines from Directive 2010/63/EU of the European Parliament on the protection of animals used for scientific purposes with institutional approval from the University of Bristol ethics committee. Male Wistar rats were euthanised by lethal injection of 140 mg/kg sodium pentobarbital (i.p.) followed by heart removal for enzymatic cell dissociation as described previously [21] and detailed in the Supplement. For Ca^2+^ imaging, ventricular cells were incubated with 5 μM Fluo-5F/AM (Thermofisher, Massachusetts, USA) then imaged on a confocal microscope (Zeiss, Oberkochen, Germany) in line-scan mode. A light emitting diode placed in the transmitted light detector pathway was used to provide precise timing information in the confocal images. Experiments were carried out at room temperature and additional experimental details are provided in the Supplement.

### 2.2 Electrophysiology

Ventricular cells were voltage-clamped with 10 mM tetraethylammonium chloride added to the 1 mM Ca^2+^ Tyrode’s solution, with a Cs^+^-based, Na^+^-free pipette solution. Cells were held at a V_m_ of −80 mV, pre-conditioned to control SR Ca^2+^ load (4 step depolarizations to 0 mV for 200 ms) and stimulated with either an AP waveform or step protocol at 0.2 Hz. For step protocols, an initial 400 ms ramp to −50 mV was used to inactivate the fast Na^+^ current. In the repolarizing step protocol (RP), V_m_ was stepped to 50 mV for 5 ms to activate LCCs, then to test potentials for 100 ms, while in the depolarizing step protocol (DP), V_m_ was stepped to test potentials for 200 ms. When indicated, 15 μM nifedipine (Sigma-Aldrich, Missouri, U.S.A.) was used to reduce LCC availability [22], so that individual Ca^2+^ sparks could be resolved [23,24].

### 2.3 Data analysis and Monte Carlo simulations

Data analysis was performed using custom computer programs written in MATLAB (v2021a, Mathworks, Massachusetts, U.S.A.). The latency of Ca^2+^ transients and Ca^2+^ sparks was measured from the start of the test pulse for DP, the end of the conditioning pulse for RP or the time at which the AP passed through −40 mV during phase 0 to the time taken for the rate of rise of fluorescence to exceed 5% of the maximum. Unless otherwise stated, error bars indicate one standard error of the mean (for experimental data), while shaded regions indicate the 95% confidence interval of the mean (for simulated data). Boxes show medians and interquartile ranges. Sample sizes are notated by n and N, which refer to the number of cells and animals, respectively. Statistical tests were performed in Graphpad Prism (v6.01) or MATLAB and a *p*-value (denoted as p) < 0.05 was considered to be the limit of statistical confidence.

Monte Carlo simulations of CICR latency incorporated stochastic LCC and RyR gating, electrodiffusion and Ca^2+^ buffering in the junctional space, and detection of Ca^2+^ by Fluo-5F and confocal microscopy. The V_m_- and Ca^2+^-dependence of LCC gating kinetics was calculated using the LCC open probability (P_O,LCC_) from existing LCC models [25,26] and mean open time (τ_O,LCC_) from single channel recordings made in near physiological conditions [27]. The V_m_-dependence of i_Ca_ was estimated using the Goldman-Hodgkin-Katz flux equation (Eq. 11, Supplementary Material) which was scaled to be −0.15 pA at −10 mV with 1 mM extracellular [Ca^2+^] [28,29]. Dyad Ca^2+^ kinetics were calculated using a model that used a quadrilateral mesh geometry and included Ca^2+^ buffering by calmodulin, ATP, Fluo-5F, troponin, mitochondria, phospholipids and RyRs. Surface charge effects due to the surface membrane (see [30]) and junctional SR membrane were included (Fig. 3F). Ca^2+^-dependent RyR gating was based on published single channel data from rat [18,31]. The simulated detection of the calculated RyR opening was delayed by ~0.3 ms due to the kinetics of Ca^2+^ binding to Fluo-5F (from the dyad Ca^2+^ model) plus the effect of microscope blurring (~0.6 ms, [32]). For Ca^2+^ release latencies where the number of initiating complexes (nIC) was >1, the latency probability distribution was adjusted for missed events by P – (1 – P)^nIC^.

## 3 Results

### 3.1 Ca^2+^ release latency during a normal action potential

**Fig. 1:**
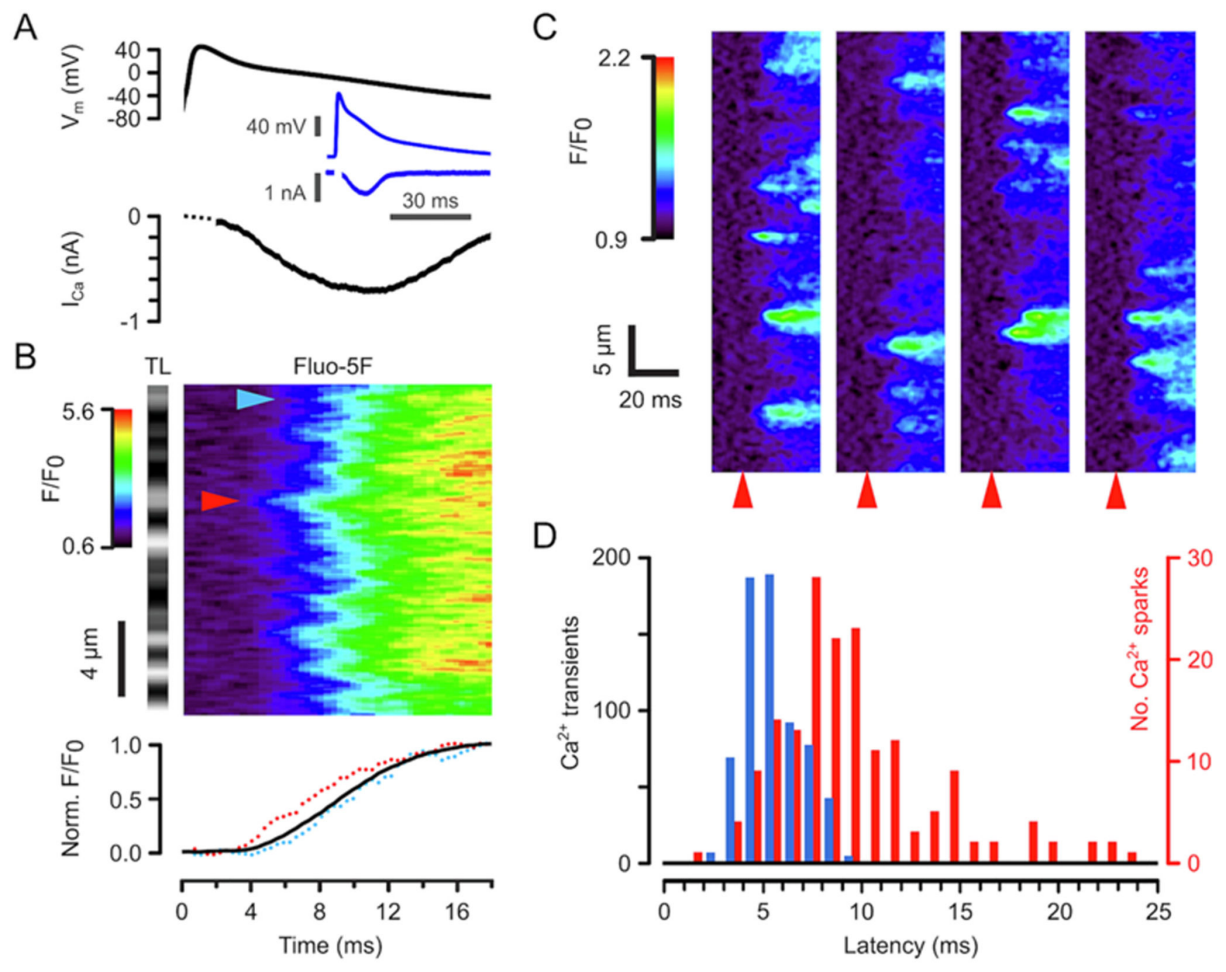
Ca^2+^ release latency during an AP. (A) Top panel shows the AP voltage-clamp waveform and the bottom panel the evoked Ca^2+^ current (nifedipine-sensitive). The insets show the same data at a slower time scale. Amplifier saturation effects during the upstroke of the AP were not completely removed by subtraction of the current recorded in nifedipine and led to an artifact at early times as indicated in grey. (B) Confocal line scan image of the normalized Fluo-5F fluorescence (F/F_0_) changes evoked by the AP shown in A. The transmitted light intensity (TL) of the line scan region is shown at the left and indicates z-line positions. The time base is the same for panels A & B. Note the high time resolution and relative uniformity in Ca^2+^ release at z-lines. The bottom panel shows the mean time course of Fluo-5F fluorescence (black line) together with local time course at the positions marked by the arrow heads. (C) Ca^2+^ sparks evoked by sequential AP clamp pulses in the presence of nifedipine. Note the much lower probability of Ca^2+^ release along the scan line and the beat-to-beat variation in position and timing. The red arrows indicate when V_m_ exceeds −40 mV during the AP upstroke. (D) Ca^2+^ spark (red bars, 170 events, n/*N* = 6/3) and Ca^2+^ transient latencies (blue bars, 721 z-lines, n/*N* = 7/3). (For interpretation of the references to colour in this figure legend, the reader is referred to the web version of this article.)

To eliminate cell-to-cell variability in action potential (AP) time course, we voltage-clamped cells using a previously recorded AP as the voltage command [33], with a low affinity fluorescent Ca^2+^ indicator (Fluo-5F) to report Ca^2+^ release. Fig. 1A (top panel) shows the early part of the AP (inset showing entire AP) with the nifedipine-sensitive Ca^2+^ current (I_Ca_) below. It is apparent from the corresponding fluorescent Ca^2+^ record, imaged at high temporal and spatial resolution (Fig. 1B), that detectable Ca^2+^ release started after a delay of ~4 ms with peak Ca^2+^ being reached in ~12 ms. The non-uniformity in the pattern of Ca^2+^ release in this line-scan image occurred with a periodicity of 1.8 μm, as expected from the resting sarcomere spacing and the diffusion of Ca^2+^ from SR release sites at z-lines (z-lines can be seen in the corresponding transmitted light image on the left of the Fluo-5F line-scan image) toward the centre of the sarcomere [2]). Variability in the time of initiation of Ca^2+^ release along the scan line is highlighted by the arrows pointing to two z-lines. The time course of the local Ca^2+^ transients at these sites are shown in the lower panel in Fig. 1B together with the average time course of the Ca^2+^ transient. These data show that the time course of Ca^2+^ release is very similar at different z-lines but differs in the latency for activation [2].

The eventual uniformity in the Ca^2+^ transient at each z-line in Fig. 1B suggests that the probability of Ca^2+^ release in the form of Ca^2+^ sparks (P_spark_) at each sarcomere must approach 1.0 [19]. This prevents individual Ca^2+^ spark latencies from being resolved since multiple Ca^2+^ release sites are likely to be within the confocal recording volume (see below and [19]). We therefore used nifedipine to block most LCCs and thereby reduce P_spark_ to the point where individual electrically stimulated events and their timing could be resolved [23,24]. Fig. 1C shows the effect of adding 15 μM nifedipine to the superfusate, where individual Ca^2+^ sparks and their latencies are clearly resolvable (time of depolarization marked by red arrows).

Fig. 1D summarises the observed Ca^2+^ release latencies in response to APs. Ca^2+^ transient (blue bars) and Ca^2+^ spark (red bars) latencies were 5.6 ± 0.1 ms (721 z-lines, n/*N* = 7/3) and 9.6 ± 0.3 ms (170 Ca^2+^ sparks, n/*N* = 6/3), respectively. At these times, the AP V_m_ was passing through 0 mV and − 12 mV (respectively). These voltages are near the peak of the V_m_-Ca^2+^ transient amplitude relationship (see below and [34]).

### 3.2 V_m_-dependence of Ca^2+^ release latency

**Fig. 2:**
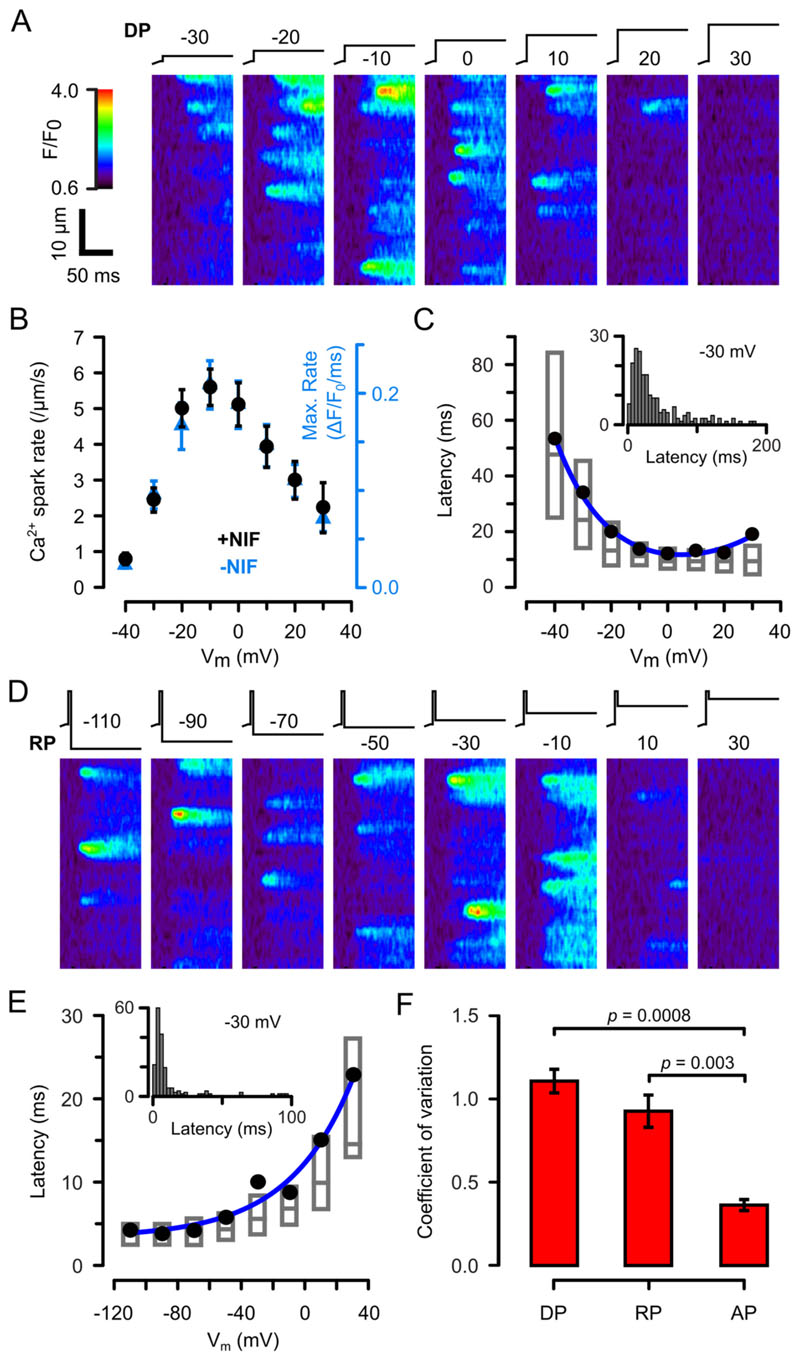
Ca^2+^ spark latencies in response to DP and RP. (A) Exemplar confocal line scan images showing Ca^2+^ sparks in the presence of nifedipine at the indicated DP V_m_. (B) Ca^2+^ spark rate during the first 20 ms (838 Ca^2+^ sparks, n/*N* = 14/6) (filled circles). The maximum rate of rise for Ca^2+^ transients for the same DP Vm is shown by blue triangles (218 Ca^2+^ transients, n/N = 14/8). There was no difference between these V_m_ dependencies when normalized (*p* > 0.999, Chi-squared test). (C) Corresponding V_m_-dependence of mean (solid circles), median and interquartile range (grey bars) Ca^2+^ spark latency (1215 Ca^2+^ sparks, n/N = 14/8). The solid blue line shows an empirical bi-exponential fit to the mean data:$\;{\text{Latency}}_{\text{DP}}=6.7\left({\text{e}}^{-\frac{\text{v}}{18.5}}+{\text{e}}^{\frac{\text{v}}{20.8}}\right)$ (r^2^ = 0.99). (D) Ca^2+^ sparks in response to RP (same cell as A). The inset shows the distribution of Ca^2+^ spark latency during DP to −30 mV. (E) V_m_-dependence of Ca^2+^ spark latency during RP in the presence of nifedipine (1201 Ca^2+^ sparks, n/*N* = 15/7). The solid blue line shows an empirical exponential fit: ${\text{Latency}}_{\text{RP}}=9.2\;{\text{e}}^{\frac{\text{v}}{39.6}}+3.3$ (r^2^ = 0.96). The inset shows the distribution of Ca^2+^ spark latency during RP to −30 mV. (F) The coefficient of variation for DP-, RP- and AP-evoked Ca^2+^ spark latencies. For DP and RP, the V_m_ were − 30 to 10 mV (*p*-values from Mann-Whitney tests). (For interpretation of the references to colour in this figure legend, the reader is referred to the web version of this article.)

To understand the observed Ca^2+^ spark latency during an AP, we examined its V_m_-dependence using a conventional depolarizing protocol (DP), where V_m_ was stepped from −50 mV to various test potentials to evoke Ca^2+^ sparks. It is apparent from the line-scan images shown in Fig. 2A that during DP, Ca^2+^ spark rate had a bell-shaped V_m_-dependence (summarised in Fig. 2B) but Ca^2+^ spark amplitude had no dependence on V_m_ (Fig. S1 and [23]). Since the maximum rate of rise of a Ca^2+^ transient (in the absence of nifedipine) should be a reasonable proxy for Ca^2+^ spark rate (Fig. 2B), the similarity of the V_m_-dependence of both (*p* > 0.999, χ^2^ test) suggests that nifedipine does not markedly alter the V_m_-dependence of LCC gating, adding confidence to the idea that latencies measured in the presence of nifedipine should reflect normal ECC. This is also consistent with the suggestion that nifedipine stabilizes the LCC in a closed state [35] (see [22] for a review).

Fig. 2C shows that the mean Ca^2+^ spark latency was ~58 ms at −40 mV and decreased with V_m_ to a minimum between −10 and 20 mV (14.5 ± 0.7 ms, 575 Ca^2+^ sparks, n/*N* = 14/6) before increasing again. This minimum DP latency was 4.9 ms longer than observed during the AP (c. f. Fig. 1D, p = 0.007, Mann-Whitney test). However, as noted above, the AP was repolarizing around the time where Ca^2+^ sparks appeared, so measuring Ca^2+^ spark latency with a conventional DP waveform may not be analogous to the physiological AP. During a pre-pulse to very positive potentials, i_Ca_ is strongly reduced and LCCs can open without activating CICR and Ca^2+^ sparks [36–38]. When V_m_ is then stepped to more negative potentials i_Ca_ increases and triggers CICR with a delay due to the kinetics of Ca^2+^ accumulation around RyRs and RyR activation kinetics. To analyse this case, we used a 5 ms 50 mV pre-pulse because, at this potential, the reduced i_Ca_ did not activate CICR (as evidenced by the lack of Ca^2+^ sparks during the pre-pulse) and Ca^2+^ influx via reverse mode Na^+^-Ca^2+^ exchange was minimal due to the absence of Na^+^ in the patch pipette filling solution.

For this repolarizing protocol (RP), Ca^2+^ sparks were evoked at all test potentials, although the number decreased at V_m_ > +10 mV (Fig. 2D). Fig. 2E shows that RP Ca^2+^ spark latency increased approximately exponentially with V_m_ (solid lines show a 40 mV e-fold increase). Below −90 mV, Ca^2+^ spark activation was very rapid with a delay that asymptotically approached 3.3 ms. This minimal latency should reflect the delay associated with RyR activation as well as diffusion and detection of Ca^2+^. At −10 mV, the RP Ca^2+^ spark latency was 8.7 ± 0.5 ms (189 Ca^2+^ sparks, n/*N* = 15/7) which is slightly (0.9 ms) shorter than observed with APs (*p* < 0.0001, Mann-Whitney test). It follows that the overshoot of the physiological AP and phase 1 repolarization serves an important role to minimise both the Ca^2+^ transient latency and non-uniformity in local Ca^2+^ release [21,39,40].

If the latency to first LCC opening were the dominant factor in the delay for Ca^2+^ spark activation, the observed Ca^2+^ spark latency distribution should be exponential. However, this was not the case (see insets Figs. 2C & E) at any potential and this observation is underscored by the median latency (bars) not being 69% of the mean latency (filled circles).

Despite the general similarity in the V_m_-dependent behaviour of Ca^2+^ sparks and Ca^2+^ transients (Fig. 2B), closer inspection of the Ca^2+^ spark initiation data showed that variability in the AP-evoked latency (as measured by the coefficient of variation, CV = σ/x^−^) was smaller than seen with RP or DP (Fig. 2F). This might seem counter-intuitive, especially if one considers that each Ca^2+^ spark occurs at a different V_m_ during the AP which should add variance due to the V_m_-dependence of Ca^2+^ spark latency. This raises the questions: How does the stochastic gating of LCCs and RyRs contribute to Ca^2+^ spark initiation and how is the variance in Ca^2+^ spark timing reduced?

### 3.3 Reconstructing the Ca^2+^ spark initiating complex

**Fig. 3:**
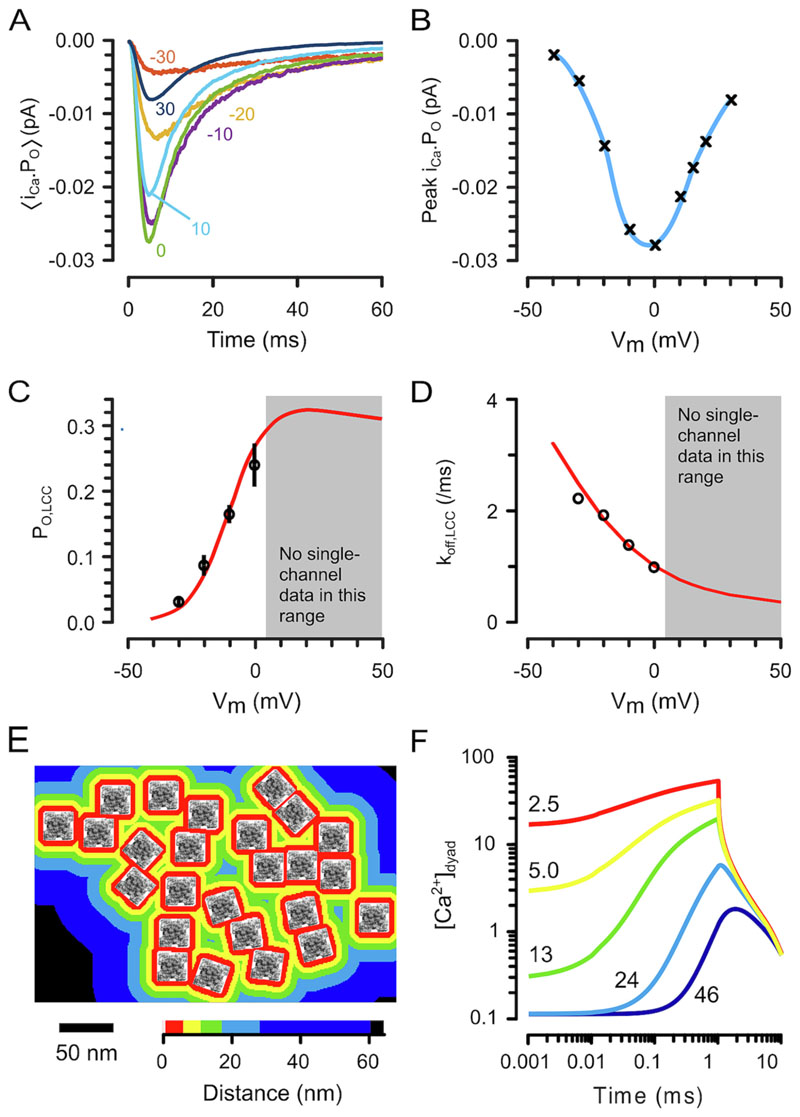
Simulation of ECC using reported LCC, RyR and dyad properties. (A) Time course of average LCC Ca^2+^ flux by DP to the V_m_ indicated (5000 simulations each). (B) V_m_-dependence of peak LCC Ca^2+^ flux from A. Note that the averaged single LCC currents in A,B are very similar to reported whole cell I_Ca_ data (e.g. [24,34]). (C) and (D) show experimental (black symbols, from Josephson et al. [27]) and model (red lines) for P_O,LCC_ and k_off,LCC_ respectively (E) A distance map for RyRs with RyR positions as shown by Asghari et al. [41]. (F) Calculated [Ca^2+^]_dyad_ 4 nm from the surface sarcolemma at the indicated lateral distances from an open LCC (i_Ca_ = 0.2 pA for 1 ms). The lateral distances shown were chosen so that these data can be directly compared Soeller & Cannell [30], as well as to the RyR distance map shown in panel E. (For interpretation of the references to colour in this figure legend, the reader is referred to the web version of this article.)

To examine the sources of variance underlying the timing of Ca^2+^ sparks, we constructed a Monte-Carlo model that calculates V_m_- and Ca^2+^-dependent LCC gating, Ca^2+^ diffusion and binding, and RyR Ca^2+‒^dependent activation rate. The model formulation (for key details, see equations in Supplement) reproduced whole-cell Ca^2+^ currents (Figs. 3A, S3) and the peak I_Ca_ I-V relationship (Fig. 3B) in accord with experimental findings. The underlying single-channel P_O,LCC_ (Fig. 3C, Eq. 9 in Supplement) and on rate (Fig. 3D, Eq. 10 in Supplement) are shown superimposed on reference single channel data [27]. The recent finding that RyR clusters may be more sparsely arranged than previously thought [41] was also incorporated. A prototypic RyR cluster, derived from published data [41], is shown in Fig. 3E and the distance from the individual RyRs is shown in colormap. Fig. 3F shows how [Ca^2+^]_dyad_, at distances shown within these colormap levels, changes in response to a 1 ms LCC opening. Shortly after the discovery of Ca^2+^ sparks, analysis of the evoked Ca^2+^ spark rate [2,3] suggested that local [Ca^2+^]_dyad_ levels would need to increase ~100-fold to explain the transition from rest to full activation. Fig. 3F shows that such levels (~10 μM) only develop (after an LCC opens) within a distance that is similar to the half-width of the RyR. This implies that the Ca^2+^ spark initiating complex (IC) cannot involve all RyRs in the cluster but only those in very close proximity to the open LCC. It follows that the low numbers of RyRs in close proximity to the open LCC should contribute the latency for Ca^2+^ spark activation. The removal of most of the latency for LCC activation during RP (compare Fig. 2C and E), along with the consequent reduction in uncertainty in LCC activation rate, would allow the Monte-Carlo model kinetic response to RP to be used to investigate the likely RyR:LCC stoichiometry within the IC.

### 3.4 Stoichiometry of Ca^2+^ spark initiating complex

**Fig. 4:**
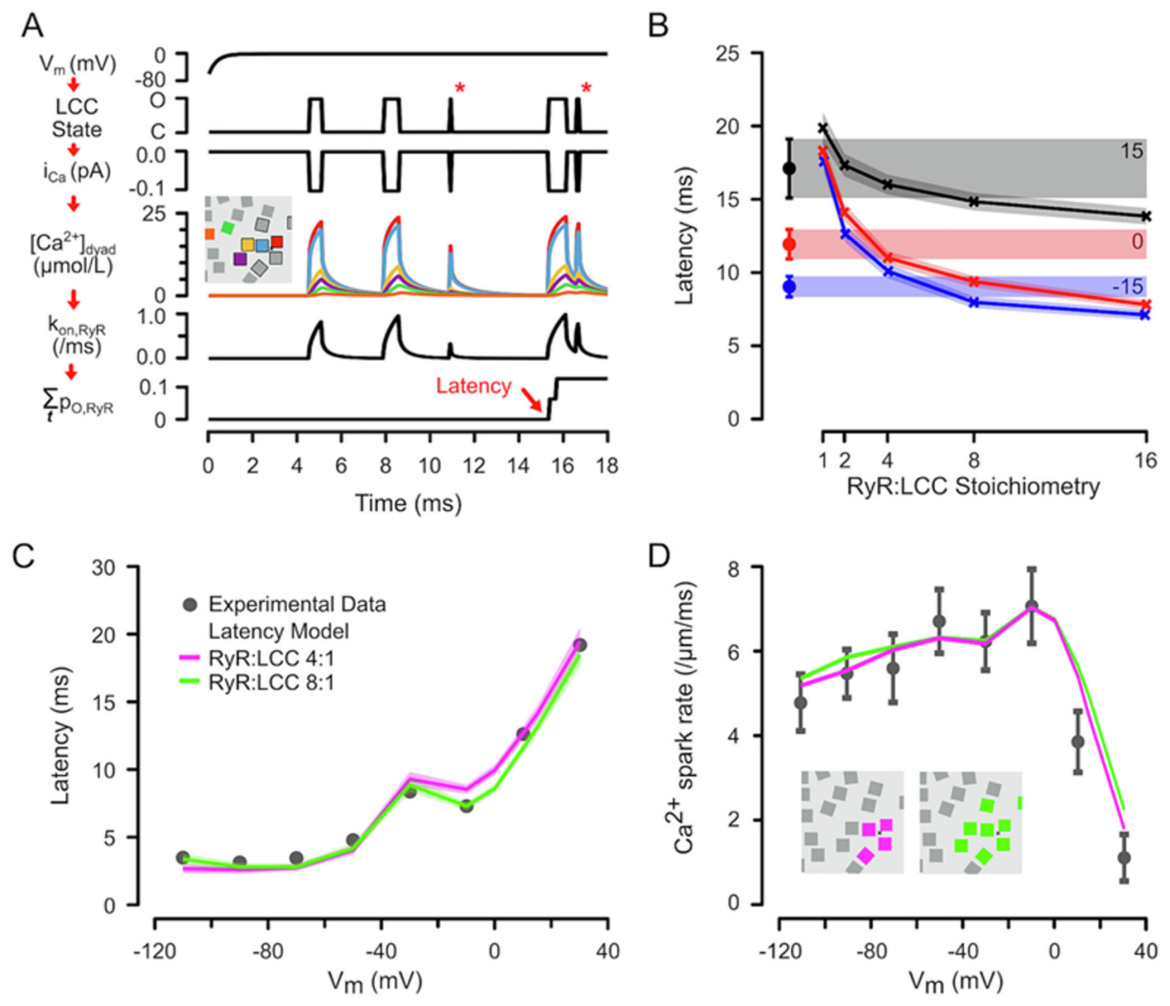
Ca^2+^ release latency during voltage-clamp steps. (A) Diagram showing modelled processes that contribute to the calculated Ca^2+^ release latency (from top to bottom): (i) DP to 0 mV with finite clamp speed (τ ~ 0.3 ms), (ii) LCC transitions from closed (‘C’) to open (‘O’) states, (iii) the corresponding Ca^2+^ influx (i_Ca_), (iv) [Ca^2+^]_dyad_ at an RyR location (indicated by colour, see inset). RyR locations were derived from electron microscopy data [41]. (v) Net RyR on rate and (vi) cumulative RyR open probability (∑p_o, RyR_), where the first opening that triggers Ca^2+^ release is indicated (red arrow). (B) Simulated mean (solid lines) and 95% confidence intervals for Ca^2+^ release latencies for various RyR:LCC ratios and RP = ‒15, 0 and 15 mV (4000 simulations for each data point). The measured latencies during RP are shown at left (from Fig. 2E). (C) Simulated V_m_-dependence of Ca^2+^ release latency for a 4:1 (purple) or 8:1 (green) RyR:LCC stoichiometry. (D) Corresponding Ca^2+^ spark rate. Inset shows RyR arrangements for the simulations. (For interpretation of the references to colour in this figure legend, the reader is referred to the web version of this article.)

As illustrated in Fig. 4A, we used the V_m_-dependence of LCC gating and corresponding i_Ca_ (Eq. 11 in Supplement) to calculate local [Ca^2+^] changes that were subject to buffering by the membranes, ATP, calmodulin and Fluo-5F. Experimental RP Ca^2+^ release latencies were then compared to the results of many simulations (1000’s) that gave the average time to activation of a model IC that had different numbers of RyRs (red arrow in lower RyR P_o_ trace in Fig. 4A). Fig. 4B shows the calculated Ca^2+^ release latency for RP at 3 potentials (chosen to reflect the V_m_ at which the majority of Ca^2+^ sparks occur during an AP, see Fig. 1D) for a range of RyR:LCC stoichiometries. Measured Ca^2+^ spark latencies at the indicated potentials are shown on the left (and extended by coloured regions across the figure) and should be compared to the behaviour of a single IC at the indicated RyR:LCC stoichiometry. At all three potentials, an RyR:LCC ratio between 3:1 and 8:1 appeared sufficient to explain the experimentally observed latency.

The V_m_-dependence of RP Ca^2+^ spark latency was reproduced with an RyR:LCC stoichiometry of 4:1 (r^2^ = 0.88) (purple, Fig. 4C) and the fit only slightly improved (r^2^ = 0.91) at 8:1 (green). Importantly, the model also fit the V_m_-dependence of Ca^2+^ spark rate reasonably well (Fig. 4D). At first sight, the slight decrease in Ca^2+^ spark rate during RP to V_m_ below ‒10 mV might appear paradoxical when i_Ca_ increases as V_m_ becomes more negative (see Eq. 11 in Supplement). However, the model showed that this can be explained by a the V_m_-dependence of LCC mean open time (τ_O,LCC_). At very negative potentials, the small τ_O,LCC_ (due to increasing deactivation) led to a decrease the average [Ca^2+^]_dyad_ associated with each LCC opening and a consequent decrease in RyR opening rate (see LCC openings marked by asterisks in Fig. 4A). When V_m_ was >10 mV Ca^2+^ spark rate decreased rapidly, reflecting the decreasing i_Ca_ and the Ca^2+^ dependence of RyR opening rate.

### 3.5 Coupling fidelity in the IC

**Fig. 5:**
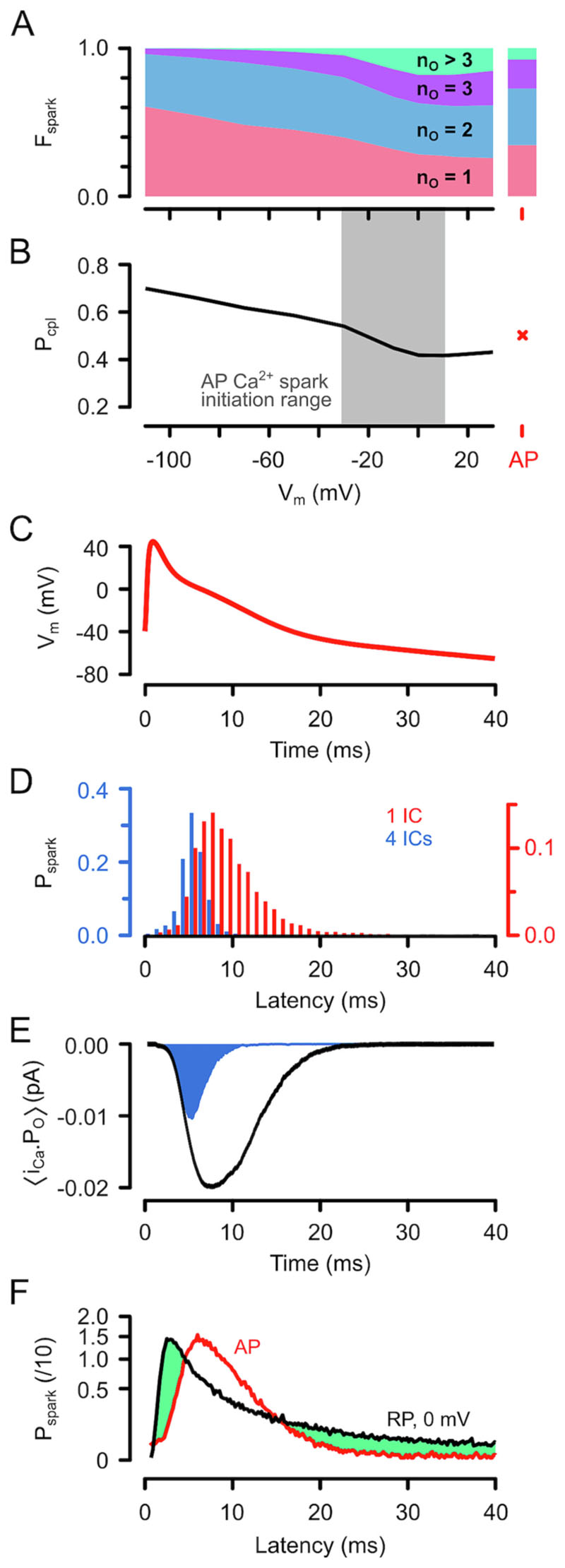
P_spark_ and P_cpl_ during RP- and AP-evoked Ca^2+^ release. (A) The fraction of Ca^2+^ sparks (F_spark_) that were evoked by n_o_ LCC openings at each V_m_ (40 00 simulations each). The right-hand side shows the corresponding F_spark_ using the AP shown in Fig. 1A. The mean n_O_ was 2.01 ± 0.01 (10,000 simulations). (B) The V_m_-dependence of coupling fidelity (P_cpl_) during RP. P_cpl_ for an AP is shown by a cross on the right. C) The exemplar AP waveform used in the computer model. D) Simulated P_spark_ using a 4:1 RyR:LCC stoichiometry for 1 (red bars) or 4 (blue bars) ICs. The mean latencies were 9.55 ± 0.06 ms (P_spark_ = 0.796, 10,000 simulations) and 5.61 ± 0.02 ms (P_spark_ = 0.997, 10,000 simulations) respectively. E) Average i_Ca_ during the AP and proportion involved in triggering Ca^2+^ transients (blue region). F) The PDFs for Ca^2+^ spark initiation during an AP (red) or RP to 0 mV (black). The results in E and F are the average of 50,000 simulations and the origin of the reduction Ca^2+^ release dyssynchrony associated with the AP compared to RP is highlighted by the green shaded regions. (For interpretation of the references to colour in this figure legend, the reader is referred to the web version of this article.)

The computer model also allows us estimate the ability of a single LCC opening to initiate a Ca^2+^ spark (“coupling fidelity”, P_cpl_ [20]). Fig. 5A shows the number of LCC openings (n_o_) associated with the initiation of each Ca^2+^ spark as a function of RP V_m_. At very negative potentials, the i_Ca_ associated with a single LCC opening (n_0_ = 1) could trigger a Ca^2+^ spark ~60% of the time. The remaining ~40% required a second LCC opening (n_0_ = 2), which is possible because not all LCCs are activated during the RP pre-pulse (note a maximum P_O,LCC_ of ~0.3 in Fig. 3C) and LCCs do not enter an absorbing closed state. Over the V_m_ range where Ca^2+^ sparks appear during the AP (Fig. 5A grey region) and in response to an AP (highlighted in red at the right, see below), Ca^2+^ sparks are most likely to occur on the second LCC opening. In summary, this analysis of n_o_ suggests that P_cpl_ decreases with V_m_ (Fig. 5B) and is 0.4–0.6 over the physiological range.

The model predictions were also tested using the AP (Fig. 5C) to give a physiological V_m_- and time-dependence to the calculated i_Ca_ and resulting Ca^2+^ spark initiation latencies. As shown in Fig. 5D, the mean simulated AP-evoked Ca^2+^ release latency in the presence of one IC was 9.55 ± 0.06 ms (red bars), which was not different to that observed experimentally (*p* = 0.55, Mann-Whitney test, cf. Fig. 1D). This is also consistent with the idea that nifedipine can reduce LCC availability to one per IC or couplon [24]. When the number of ICs in the dyad was increased to 4, the latency for Ca^2+^ release was approximately halved and was not different to that observed for Ca^2+^ transients (*p* = 0.24, Mann-Whitney test, cf. Fig. 1D). Fig. 5E shows the simulated average i_Ca_ during an AP (black line), with the fraction that was responsible for Ca^2+^ transient activation in the blue shaded region and comprised ~20% of the integrated i_Ca_. Fig. 5F shows the probability density functions for Ca^2+^ spark latency during the AP or RP to 0 mV and explains how the variability in Ca^2+^ release latency is reduced by the AP waveform. At a latencies <4.5 ms, P_spark_ is higher for RP because V_m_ during the AP is > +10 mV, which reduces P_spark_ and increases latency (see Fig. 2E). At latencies >15 ms, P_spark_ for the AP is also reduced compared to RP because LCCs are deactivating. The net effect is that the P_spark_ probability density function over time is narrower than for RP, resulting in less variability in Ca^2+^ spark timing (CV = 0.56 and 1.21 for AP and RP to 0 mV, respectively) and a hence a more spatially uniform Ca^2+^ transient.

## 4 Discussion

The experiments and modelling shown here provide quantitative insight into the control of CICR during APs. While previous studies have shown that AP phase 1 repolarization can increase Ca^2+^ release synchrony [21,39,40], this effect has been ascribed solely to increasing i_Ca_ during phase 1. The present study shows that explanation is incomplete because the kinetics of LCC gating also plays a role during early and later repolarization (Fig. 5F). So, while RP, which can mimic a very fast and deep AP phase 1, can reduce latency compared to DP it does not, by itself, produce the same degree of synchrony in Ca^2+^ release as an AP. The overshoot (i.e. V_m_ > 0 mV) of the AP also reduces variance in the timing of CICR and this is central to the production of a more uniform Ca^2+^ transient. The new latency data (and their empirical equation fits) presented here also form a “ground truth” for the development of future models of cardiac ECC which may find application within in-silico drug screening, and the present computer model illustrates how these data can be used to gain new insight into the operation of CICR at the single channel level.

### 4.1 Definition of the Ca^2+^ spark initiation complex

Previous work has shown that a single LCC can initiate a Ca^2+^ spark at negative potentials [23,24,42]. However, such experiments did not give insight into the number of LCCs, P_cpl_ or the number of local RyRs involved in Ca^2+^ spark initiation at physiological potentials. By examining the V_m_-dependence of Ca^2+^ spark latency and fitting a detailed model to observed behaviour we have found that the latency for Ca^2+^ sparks during the AP can be explained by 4 ICs (at a minimum) each made up of an LCC and at least 3 RyRs. This stoichiometry provides the first functional definition of the physiological couplon, with the local group of RyRs in the IC being most likely to be activated on the second opening of the nearby LCC. The simulations also showed that the apparent RyR:LCC stoichiometry increases as V_m_ becomes more negative (Fig. 4B), an effect that is due to the increase in i_Ca_ activating a larger group of RyRs, whose summed opening rate decreases latency.

Only ~20% of the total LCC current seems to be needed to trigger CICR, and within the parlance of CICR theory forms the “triggering” component of I_Ca_ [43]. The remaining excess ensures activation of every couplon during the AP and also provides additional Ca^2+^ influx to control SR Ca^2+^ content and releasable Ca^2+^. The fraction of I_Ca_ responsible for triggering Ca^2+^ sparks is sensitive to the AP waveform due to the interactions between V_m_, i_Ca_ and LCC gating. A prolongation of AP phase 1 may increase or decrease net Ca^2+^ influx via I_Ca_ (depending on the exact AP time course). However, an increase in the time taken for i_Ca_ to increase to the point where Ca^2+^ spark initiation is likely will increase Ca^2+^ spark latency (see Fig. S4) and reduce P_cpl_.

Typically, two LCC openings (P_cpl_ ~ 0.5) were needed to trigger CICR to reproduce the observed mean Ca^2+^ spark latency (which was greater than the mean closed of the LCC) (Fig. 5A). While the number of LCCs needed to produce the observed delay is predicated on the closed time of the LCC, we were constrained by available LCC gating data [27]. The possibility of modal [44] or cooperative gating [45] in LCCs is problematic since these effects could increase the effective closed time and would have prevented the simple LCC gating model from fitting the observed V_m_-dependence of Ca^2+^ spark latency. Since this was not the case, such gating effects may be limited to a subset of clusters at the limits of the V_m_ range studied (see below) and/or under different conditions. Nevertheless, the finding that P_cpl_ ~ 0.5 should not be changed by such effects. It is important to note that, while it is the activation of the first IC that leads to a Ca^2+^ spark, the other ICs (and their RyRs) serve to increase the Ca^2+^ release flux when they are activated (either by their nearest LCC or by Ca^2+^ release from the triggering IC).

### 4.2 Comparison to binding, imaging and biophysical studies

The requirement for an RyR:LCC stoichiometry of 3–8:1 (Fig. 4B) per IC with up to 4 ICs per couplon is compatible with binding studies [46,47] and flux comparisons [48] that indicate a RyR:LCC stoichiometry of ~7:1. Our data is also compatible with super-resolution imaging that suggests a mean of ~8–13 RyRs per “cluster” [49,50] and 2 clusters per couplon [50]. The convergence of these values obtained by very different techniques argues against very large numbers of RyRs in a couplon and we note that previous confocal [51] and electron microscopy quantification [6] would have over-estimated the number of RyRs due to the assumption of tight RyR packing. Correcting the previous optical estimate of 78 RyRs/couplon [51] for a packing density of 40% [41] yields ~30 RyRs/couplon. Similar correction of electron microscopy data [52] suggests 23 RyRs per couplon. Another estimate of the number of RyRs in a couplon was provided by noise analysis [19] and suggested >18 RyRs per couplon, while a peak flux during a Ca^2+^ spark of 7.9 pA [32] with a single RyR channel current of 0.5 pA [53,54] and P_O_ of 0.5–0.8 [18,31] suggest ~24 RyRs per couplon. Together, these estimates seem to converge toward a mean of ~23 RyRs per couplon, which is in good agreement with the data presented here.

### 4.3 Coupling fidelity

Some previous studies have suggested that P_cpl_ is much lower (<0.05, [20,38,55]) than estimated here, although the reported value is dependent on the experimental protocol. Uncertainty in the reported P_cpl_ may arise from extrapolation to physiological conditions (e.g. the assumed V_m_-dependence of i_Ca_ and τ_O,LCC_, and the τ_O,LCC_-dependence of P_spark_). With a very low P_cpl_, a high P_spark_ (~1.0) during an AP becomes problematic and requires large numbers of LCCs (25–600, [20,38,55]) to be available in the couplon, and such numbers may not be compatible with electrophysiological and binding data. Our data and analysis remove this problem: with P_cpl_ = 0.5 and ~ 4 ICs in each couplon, P_spark_ is 0.94 for each LCC opening and with the assumption of 2 openings during the AP (see Fig. 5A) P_spark_ becomes 0.99.

It is important to note that P_cpl_ is not the same as the ability of the LCC Ca^2+^ flux to (eventually) trigger Ca^2+^ release. Thus, experiments that modulate i_Ca_ [42,56] and which have shown that LCCs can trigger a Ca^2+^ sparks with high fidelity, are consistent with P_cpl_ as defined here.

### 4.4 Limitations of the model

Despite the ability of the model to accurately reproduce the V_m_-dependence of RP Ca^2+^ spark latency (Fig. 3C), the fit to DP Ca^2+^ spark latency at positive potentials developed a systematic error (see Fig. S2). We suggest this is due to a deficiency in the LCC gating model at higher potentials since DP Ca^2+^ spark latency is highly dependent on the LCC closed and open times. Unfortunately, we could not find single channel gating data in this V_m_ range that could be used to further refine model LCC behaviour. Nevertheless, the model reproduced a clear inflexion in the RP Ca^2+^ spark latency curve (Fig. 3C) around ‒10 mV which adds to confidence in the overall behaviour of the model. This inflexion was due to interactions between the V_m_- and Ca^2+^-dependence of LCC inactivation, as well as the V_m_-dependence of P_cpl_.

### 4.5 Physiological implications

There should be an optimal RyR:LCC stoichiometry for the IC and the number of ICs in a couplon. Our simulations showed that the steep gradients of Ca^2+^ within the dyad (Fig. 3F), transduced via the nonlinear RyR opening rate resulted in strong constraints on the number of RyRs in an IC (c.f. Fig. 4B) and ~5 RyRs:1 LCC per IC and 4 ICs per couplon was sufficient to explain both P_spark_ as well as Ca^2+^ spark latency. This stoichiometry not only minimises latency, but also latency variance during the AP to produce a more uniform Ca^2+^ transient. Adding more LCCs to an IC produces redundant Ca^2+^ influx at later times and this extra Ca^2+^ has energetic consequences because it has to be pumped out of the cell (eventually). In addition, a decreased delay to first LCC opening does not translate to a similar reduction in Ca^2+^ release latency because i_Ca_ is reduced at earlier times due to the reduced driving force for Ca^2+^ entry. Adding more RyRs does not markedly increase P_cpl_ because each additional RyR has to be placed further from the initiating LCC and will be exposed to a lower local [Ca^2+^] when the LCC opens (see Fig. 3F). In addition, the release flux from the couplon is likely limited by local SR Ca^2+^ depletion [18] so additional RyRs may not increase the overall CICR amplification but will increase the risk of arrhythmogenic spontaneous Ca^2+^ sparks during diastole. While dispersion of RyRs in heart failure [49,57] could reduce the number of RyRs in an IC, P_cpl_ should not change markedly until their mean distance from the LCC is increased significantly. On the other hand, dispersion of functional ICs should lead to a more inhomogeneous Ca^2+^ transient and may explain the recent observation of “wandering” Ca^2+^ sparks [58] and compound the problem of IC uncoupling by t-tubule loss [59].

## Supplementary Material

Supplementary data to this article can be found online at https://doi.org/10.1016/j.yjmcc.2023.07.005.

Supplementary Material

## Data Availability

The data underlying this article will be shared on reasonable request to the authors.

## Abbreviations

AP: action potential
CICR: Ca^2+^-induced Ca^2+^ release
DP: depolarizing step voltage protocol
ECC: excitation-contraction coupling
IC: initiating complex
i_Ca_: unitary L-type Ca^2+^ current
I_Ca_: L-type Ca^2+^ current
LCC: L-type Ca^2+^ channel
P_cpl_: coupling fidelity
P_O,LCC_: LCC open probability
P_spark_: evoked Ca^2+^ spark probability
RP: repolarizing step voltage protocol
RyR: ryanodine receptor (SR Ca^2+^ release channel)
SR: sarcoplasmic reticulum
τ_O,LCC_: LCC mean open time
V_m_: membrane potential

## References

[1] Fabiato A, Fabiato F (1975). Contractions induced by a calcium-triggered release of calcium from the sarcoplasmic reticulum of single skinned cardiac cells. J Physiol.

[2] Cannell MB, Cheng H, Lederer WJ (1994). Spatial non-uniformities in [Ca^2+^]_i_ during excitation-contraction coupling in cardiac myocytes. Biophys J.

[3] Wier WG, Egan TM, López-López JR, Balke CW (1994). Local control of excitation-contraction coupling in rat heart cells. J Physiol.

[4] Sutko JL, Kenyon JL (1983). Ryanodine modification of cardiac muscle responses to potassium-free solutions. Evidence for inhibition of sarcoplasmic reticulum calcium release. J Gen Physiol.

[5] Lai FA, Anderson K, Rousseau E, Liu Q-Y, Meissner G (1988). Evidence for a Ca^2+^ channel within the ryanodine receptor complex from cardiac sarcoplasmic reticulum. Biochem Biophys Res Commun.

[6] Franzini-Armstrong C, Protasi F, Ramesh V (1999). Shape, size, and distribution of Ca^2+^ release units and Couplons in skeletal and cardiac muscles. Biophys J.

[7] Jorgensen AO, Shen AC, Arnold W, McPherson PS, Campbell KP (1993). The Ca^2+^-release channel/ryanodine receptor is localized in junctional and corbular sarcoplasmic reticulum in cardiac muscle. J Cell Biol.

[8] Stern MD (1992). Theory of excitation-contraction coupling in cardiac muscle. Biophys J.

[9] Cheng H, Lederer WJ, Cannell MB (1993). Calcium sparks: elementary events underlying excitation-contraction coupling in heart muscle. Science.

[10] Cannell MB, Kong CHT (2012). Local control in cardiac E–C coupling. J Mol Cell Cardiol.

[11] Sobie EA, Ramay HR (2009). Excitation-contraction coupling gain in ventricular myocytes: insights from a parsimonious model: EC coupling gain model. J Physiol.

[12] Agrawal A, Wang K, Polonchuk L, Cooper J, Hendrix M, Gavaghan DJ, Mirams GR, Clerx M (2023). Models of the cardiac L-type calcium current: a quantitative review. WIREs Mech Dis.

[13] Tanskanen AJ, Greenstein JL, Chen A, Sun SX, Winslow RL (2007). Protein geometry and placement in the cardiac dyad influence macroscopic properties of calcium-induced calcium release. Biophys J.

[14] Koh X, Srinivasan B, Ching HS, Levchenko A (2006). A 3D Monte Carlo analysis of the role of dyadic space geometry in spark generation. Biophys J.

[15] Iaparov BI, Zahradnik I, Moskvin AS, Zahradníková A (2021). In silico simulations reveal that RYR distribution affects the dynamics of calcium release in cardiac myocytes. J Gen Physiol.

[16] Xie Y, Yang Y, Galice S, Bers DM, Sato D (2019). Size matters: ryanodine receptor cluster size heterogeneity potentiates calcium waves. Biophys J.

[17] Williams GSB, Smith GD, Sobie EA, Jafri MS (2010). Models of cardiac excitation–contraction coupling in ventricular myocytes. Math Biosci.

[18] Laver DR, Kong CHT, Imtiaz MS, Cannell MB (2013). Termination of calcium-induced calcium release by induction decay: an emergent property of stochastic channel gating and molecular scale architecture. J Mol Cell Cardiol.

[19] Bridge JHB, Ershler PR, Cannell MB (1999). Properties of Ca^2+^ sparks evoked by action potentials in mouse ventricular myocytes. J Physiol.

[20] Zhou Y-Y, Song L-S, Lakatta EG, Xiao R-P, Cheng H (1999). Constitutive β_2_-adrenergic signalling enhances sarcoplasmic reticulum Ca^2+^ cycling to augment contraction in mouse heart. J Physiol.

[21] Cooper PJ, Soeller C, Cannell MB (2010). Excitation–contraction coupling in human heart failure examined by action potential clamp in rat cardiac myocytes. J Mol Cell Cardiol.

[22] McDonald TF, Pelzer S, Trautwein W, Pelzer DJ (1994). Regulation and modulation of calcium channels in cardiac, skeletal, and smooth muscle cells. Physiol Rev.

[23] Collier ML, Thomas AP, Berlin JR (1999). Relationship between L-type Ca^2+^ current and unitary sarcoplasmic reticulum Ca^2+^ release events in rat ventricular myocytes. J Physiol.

[24] Santana LF, Cheng H, Gómez AM, Cannell MB, Lederer WJ (1996). Relation between the sarcolemmal Ca ^2+^ current and Ca ^2+^ sparks and local control theories for cardiac excitation-contraction coupling. Circ Res.

[25] O’Hara T, Virág L, Varró A, Rudy Y (2011). Simulation of the undiseased human cardiac ventricular action potential: model formulation and experimental validation. PLoS Comput Biol.

[26] Grandy SA, Howlett SE (2006). Cardiac excitation-contraction coupling is altered in myocytes from aged male mice but not in cells from aged female mice. Am J Physiol-Heart Circ Physiol.

[27] Josephson IR, Guia A, Sobie EA, Lederer WJ, Lakatta EG, Stern MD (2010). Physiologic gating properties of unitary cardiac L-type Ca^2+^ channels. Biochem Biophys Res Commun.

[28] Guia A, Stern MD, Lakatta EG, Josephson IR (2001). Ion concentration-dependence of rat cardiac unitary L-type Calcium Channel conductance. Biophys J.

[29] Rose WC, Balke CW, Wier WG, Marban E (1992). Macroscopic and unitary properties of physiological ion flux through L-type Ca^2+^ channels in guinea-pig heart cells. J Physiol.

[30] Soeller C, Cannell MB (1997). Numerical simulation of local calcium movements during L-type calcium channel gating in the cardiac diad. Biophys J.

[31] Fill M, Gillespie D (2018). Ryanodine receptor open times are determined in the closed state. Biophys J.

[32] Kong CHT, Laver DR, Cannell MB (2013). Extraction of sub-microscopic Ca fluxes from blurred and noisy fluorescent Indicator images with a detailed model fitting approach. PLoS Comput Biol.

[33] Doerr T, Denger R, Trautwein W (1989). Calcium currents in single SA nodal cells of the rabbit heart studied with action potential clamp. Pflugers Arch.

[34] Cannell MB, Berlin JR, Lederer WJ (1987). Effect of membrane potential changes on the calcium transient in single rat cardiac muscle cells. Science.

[35] Hadley RW, Lederer WJ (1995). Nifedipine inhibits movement of cardiac calcium channels through late, but not early, gating transitions. Am J Physiol-Heart Circ Physiol.

[36] Isenberg G, Han S (1994). Gradation of Ca^2+^-induced Ca^2+^ release by voltage-clamp pulse duration in potentiated guinea-pig ventricular myocytes. J Physiol.

[37] Cannell MB, Cheng H, Lederer WJ (1995). The control of calcium release in heart muscle. Science.

[38] Poláková E, Zahradníková A, Pavelková J, Zahradník I, Zahradníková A (2008). Local calcium release activation by DHPR calcium channel openings in rat cardiac myocytes: local calcium release activation in cardiac myocytes. J Physiol.

[39] Sah R, Ramirez RJ, Kaprielian R, Backx PH (2001). Alterations in action potential profile enhance excitation-contraction coupling in rat cardiac myocytes. J Physiol.

[40] Fowler ED, Wang N, Hezzell MJ, Chanoit G, Hancox JC, Cannell MB (2022). Improved Ca^2+^ release synchrony following selective modification of I_tof_ and phase 1 repolarization in normal and failing ventricular myocytes. J Mol Cell Cardiol.

[41] Asghari P, Scriven DR, Ng M, Panwar P, Chou KC, van Petegem F, Moore ED (2020). Cardiac ryanodine receptor distribution is dynamic and changed by auxiliary proteins and post-translational modification. ELife.

[42] Altamirano J, Bers DM (2007). Voltage Dependence of Cardiac Excitation–Contraction Coupling: Unitary Ca^2+^ Current Amplitude and Open Channel Probability. Circ Res.

[43] Fabiato A (1985). Simulated calcium current can both cause calcium loading in and trigger calcium release from the sarcoplasmic reticulum of a skinned canine cardiac Purkinje cell. J Gen Physiol.

[44] Hess P, Lansman JB, Tsien RW (1984). Different modes of ca channel gating behaviour favoured by dihydropyridine Ca agonists and antagonists. Nature.

[45] Sato D, Dixon RE, Santana LF, Navedo MF (2018). A model for cooperative gating of L-type Ca^2+^ channels and its effects on cardiac alternans dynamics. PLoS Comput Biol.

[46] Wibo M, Bravo G, Godfraind T (1991). Postnatal maturation of excitation-contraction coupling in rat ventricle in relation to the subcellular localization and surface density of 1,4-dihydropyridine and ryanodine receptors. Circ Res.

[47] Bers DM, Stiffel VM (1993). Ratio of ryanodine to dihydropyridine receptors in cardiac and skeletal muscle and implications for E-C coupling. Am J Physiol-Cell Physiol.

[48] Wang S-Q, Song L-S, Lakatta EG, Cheng H (2001). Ca^2+^ signalling between single L-type Ca^2+^ channels and ryanodine receptors in heart cells. Nature.

[49] Sheard TMD, Hurley ME, Colyer J, White E, Norman R, Pervolaraki E, Narayanasamy KK, Hou Y, Kirton HM, Yang Z, Hunter L (2019). Three-dimensional and chemical mapping of intracellular signaling nanodomains in health and disease with enhanced expansion microscopy. ACS Nano.

[50] Shen X, van den Brink J, Hou Y, Colli D, Le C, Kolstad TR, MacQuaide N, Carlson CR, Kekenes-Huskey PM, Edwards AG, Soeller C (2019). 3D dSTORM imaging reveals novel detail of ryanodine receptor localization in rat cardiac myocytes. J Physiol.

[51] Soeller C, Crossman D, Gilbert R, Cannell MB (2007). Analysis of ryanodine receptor clusters in rat and human cardiac myocytes. Proc Natl Acad Sci.

[52] Hayashi T, Martone ME, Yu Z, Thor A, Doi M, Holst MJ, Ellisman MH, Hoshijima M (2009). Three-dimensional electron microscopy reveals new details of membrane systems for Ca^2+^ signaling in the heart. J Cell Sci.

[53] Kettlun C, González A, Ríos E, Fill M (2003). Unitary Ca^2+^ current through mammalian cardiac and amphibian skeletal muscle ryanodine receptor channels under near-physiological ionic conditions. J Gen Physiol.

[54] Gillespie D, Fill M (2008). Intracellular calcium release channels mediate their own countercurrent: the ryanodine receptor case study. Biophys J.

[55] Cheng H, Lederer WJ (2008). Calcium Sparks. Physiol Rev.

[56] Inoue M, Bridge JHB (2005). Variability in couplon size in rabbit ventricular myocytes. Biophys J.

[57] Kolstad TR, van den Brink J, MacQuaide N, Lunde PK, Frisk M, Aronsen JM, Norden ES, Cataliotti A, Sjaastad I, Sejersted OM, Edwards AG (2018). Ryanodine receptor dispersion disrupts Ca^2+^ release in failing cardiac myocytes. ELife.

[58] Hou Y, Laasmaa M, Li J, Shen X, Manfra O, Nordén ES, Le C, Zhang L, Sjaastad I, Jones PP, Soeller C (2023). Live-cell photoactivated localization microscopy correlates nanoscale ryanodine receptor configuration to calcium sparks in cardiomyocytes. Nat Cardiovasc Res.

[59] Dibb KM, Louch WE, Trafford AW (2022). Cardiac transverse tubules in physiology and heart failure. Annu Rev Physiol.

