# Supplementary material for: Ca2+ spark latency and control of intrinsic Ca2+ release dyssynchrony in rat cardiac ventricular muscle cells
Source: J Mol Cell Cardiol. Author manuscript; Available in PMC 2024 Oct 7. (PMC7616665; doi:10.1016/j.yjmcc.2023.07.005)
Supplement: Supplementary Material [file EMS198481-supplement-Supplementary_Material.pdf]

Supplementary Materials

Supplementary Methods

Preparation of isolated myocytes

After heart removal, it was Langendorff perfused with an oxygenated modified Ca^{2+}-free Tyrode's solution (in mM): 140 NaCl, 4 KCl, 1 $MgCl_2$, 10 HEPES, 10 D-glucose, pH = 7.4 at 37 °C for 5 min, followed by the same solution with 200 μM $CaCl_2$, collagenase II (1 mg/mL Worthington Biochemical Corp., New Jersey, U.S.A.) and protease I (0.1 mg/mL, Sigma Aldrich, Missouri, U.S.A.) for ~15 min. The ventricles were then cut off the apparatus and minced in 100 μM Ca^{2+} Tyrode's solution. The [Ca^{2+}] in the resulting cell suspension was gradually raised to 0.5 or 1 mM over ~10 mins and cells were stored at room temperature (RT) until used.

Ca^{2+} imaging

Cells were incubated with 5 μM Fluo-5F AM (Thermofisher, Massachusetts, U.S.A.) for 25 min at RT. The imaging chamber used a no. 1.5 glass cover-slip bottom and was mounted on an inverted LSM 710 or 880 confocal microscope (Zeiss, Oberkochen, Germany) fitted with a 40x 1.1 NA water immersion objective. Line-scan images of Ca^{2+} along the cell long axis were recorded at 0.3-0.6 ms/line and 0.083 µm/pixel.

Electrophysiology

Cells were voltage-clamped using a VE-2 amplifier and headstage (Alembic Instruments Inc., Montreal, Canada) with 90% series resistance compensation. Pipettes were pulled from borosilicate glass capillaries (Harvard Instruments, Massachusetts, U.S.A.) on a horizontal puller (P-87, Sutter Instrument Co., California, U.S.A.) and filled with (in mM): 100 Cs-Aspartate, 30 CsCl, 5 MgATP, 10 HEPES, 0.1 Fluo-5F pentapotassium salt, pH = 7.2 (with CsOH) to give resistances of 1.5-2 MΩ. Reverse mode NCX was minimised by the absence of Na^{+} in the pipette solution. During recording, cells were superfused with 1 mM Ca^{2+} Tyrode's solution that also contained 10 mM tetraethylammonium chloride.

AP-clamp was employed to reduce cell-to-cell variability in AP time-course. The AP used as the command profile for AP-clamp experiments was measured using an Axopatch 200B amplifier and CV201A headstage (Molecular Devices, California, U.S.A.) in current-clamp mode. The pipette solution for these recordings was 140 mM KCl, pH = 7.2.

Data analysis

Analysis of current recordings and line-scan images and computer simulations were performed using custom programs written in MATLAB (2021a, Massachusetts, U.S.A.). A junction potential of 10 mV was corrected during analysis. Peak I_{Ca} was calculated by subtracting the end-of-pulse current from the peak inward current. Error bars indicate one standard error of mean (S.E.M.). Unless otherwise stated, voltage values are given in mV and $[Ca^{2+}]$ in µM.

To determine the start of a Ca^{2+} release event, the rising phase was fitted to a Boltzmann equation and the start of SR Ca^{2+} release was defined as where the fitted curve exceeded 10% of the maximum rate of rise.

Monte Carlo simulations of SR Ca^{2+} release latency

The V_m-dependence of LCC open probability ($P_{O,LCC}$) was determined by previously-published models [1,2] (see Eqns. 1-9). The rate of change of a gating parameter, x, is given by Eqn. 1, where x_∞ and x_τ denote the steady-state value and time-constant, respectively. Using this format, the activation (d) and V_m-dependent inactivation (f) parameters are described by Eqns. 2-3 and 4-7, respectively. The rate of Ca^{2+}-dependent inactivation (fCa) is described by Eqn. 8.

$$\frac{dx}{dt}=\frac{x_\infty-x}{x_\tau} \quad \text{Eqn. 1}$$

$$d_\infty(V_m)=\frac{1.0}{1.0+e^{-\frac{(V_m+11)}{6.0}}} \quad \text{Eqn. 2}$$

$$d_\tau(V_m)=\left[\frac{3.0}{\left(1+e^{-\frac{(V_m+50)}{16}}\right)}+0.2\right]\cdot\left[\frac{0.49}{\left(1+e^{\frac{(V_m+2)}{10}}\right)}+0.51\right] \quad \text{Eqn. 3}$$

$$f_\infty(V_m)=\frac{1.0}{1.0+e^{\frac{(V_m+30)}{7.5}}} \quad \text{Eqn. 4}$$

$$f_{\tau,fast}(V_m)=\frac{1.0}{2.25\times10^{-3}\cdot\left(e^{-\frac{V_m+20}{10}}+e^{\frac{V_m+20}{10}}\right)}+14 \quad \text{Eqn. 5}$$

$$f_{\tau,slow}(V_m)=\frac{1.0}{2\times10^{-3}\cdot\left(e^{-\frac{V_m+5}{4}}+e^{\frac{V_m+5}{6}}\right)}+35$$ Eqn. 6

$$f_{\tau}(v)=0.7\cdot f_{\tau,fast}+0.3\cdot f_{\tau,slow}$$ Eqn. 7

$$\frac{dfCa}{dt}([Ca^{2+}]_{dyad,LCC})=\frac{1.7}{1.0+\left(\frac{100}{[Ca^{2+}]_{dyad,LCC}}\right)}$$ Eqn. 8

$$P_{O,LCC}(V_m,[Ca^{2+}]_{dyad,LCC},t)=0.5\cdot d\cdot f\cdot fCa$$ Eqn. 9

$P_{O,LCC}$ was calculated using Eqn. 9, scaled so that $P_{O,LCC}$ matched that measured in near physiological conditions [3]. The open time ($\tau_{O,LCC}$, Eqn. 10) was then used to determine the V_m-dependence of closed times ($\tau_{C,LCC}$).

$$\tau_{O,LCC}(V_m)=\frac{3}{1.0+e^{-\frac{V_m-20}{21}}}+0.15$$ Eqn. 10

The V_m-dependence of unitary LCC Ca flux, i_{Ca}, was:

$$i_{Ca}(V_m)=1.76\times10^{-5}\cdot\left(\frac{[Ca^{2+}]_{dyad,LCC}-0.341\cdot1mM\cdot e^{-\frac{2FV_m}{RT}}}{1-e^{-\frac{2FV_m}{RT}}}\right)$$ Eqn. 11

where R is the Universal Gas constant, F the Faraday and T temperature in Kelvin. This model was able to reproduce whole-cell (nifedipine-sensitive) LCC Ca^{2+} influx (Fig. 3A) and I-V relation (Fig. 3B) and.

The shape of an array of RyRs inside the dyad was obtained from electron microscopy data [4] and LCCs were placed 10-12 nm from the nearest RyRs using a Delaunay Triangulation method as illustrated in Fig. 3E. RyR gating was based on a 2-state model where the rate constants were derived from a composite of published single rat RyR channel experiments [5,6]. The key Ca^{2+}-dependence of RyR channel on rate ($k_{on,RyR}$) was:

$$k_{on,RyR}\left([Ca^{2+}]_{dyad,RyR}\right) = \frac{1}{1+\left(\frac{40}{[Ca^{2+}]_{dyad,RyR}}\right)^2} \quad \text{Eqn. 12}$$

Supplementary Figures

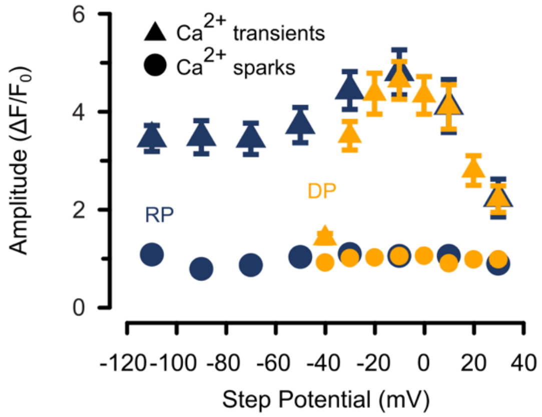

Fig. S1. Amplitude of Ca^{2+} transients and Ca^{2+} sparks evoked by RP or DP. Sample sizes are the same as for Fig. 2.

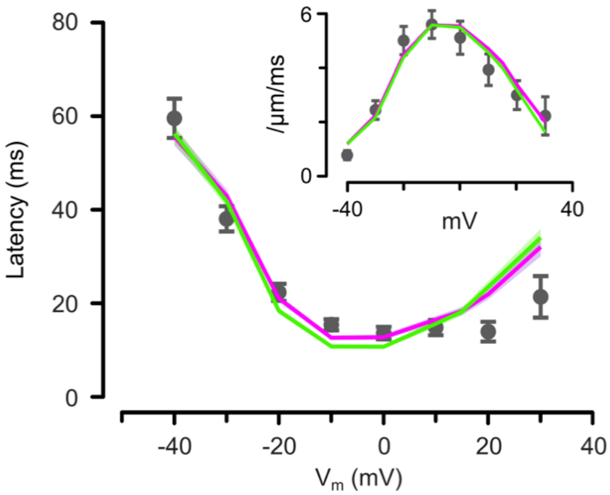

Fig. S2. Simulated V_m-dependence of Ca^{2+} spark latency during DP for 4:1 (purple) and 8:1 (green) RyR:LCC stoichiometries. Experimental data is superimposed (solid circles). Inset shows simulated Ca^{2+} spark rate (/µm/ms).

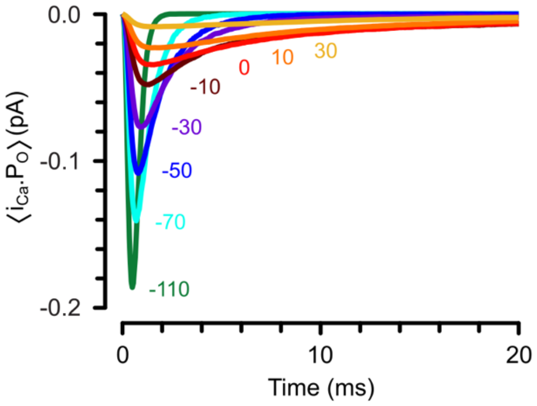

Fig. S3. Time-course of average LCC Ca^{2+} flux by RP to the indicated V_m (5000 simulations each).

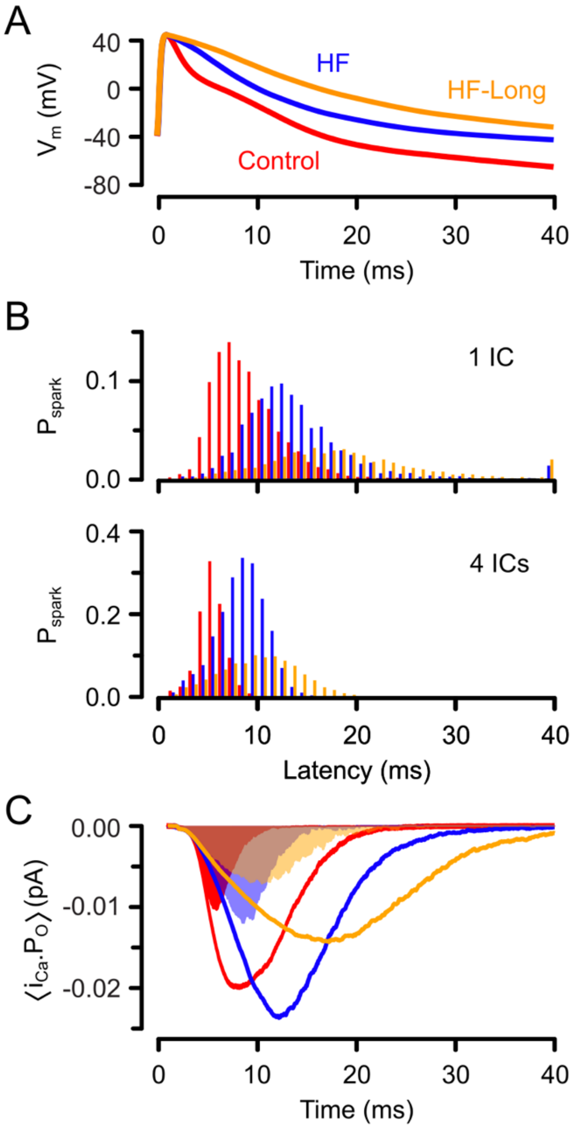

Fig. S4. Effect of AP waveform on Ca^{2+} release latency. A) Time-course of an AP from a post myocardial infarction rat model of heart failure (HF, blue line, from [7]) superimposed on the control AP from Fig. 5. The peak V_m and initial upstroke have been matched to avoid confounding results. Due to variability in the literature (e.g. [8]), an even longer AP (HF-long, yellow line) has also been used. B) Simulated P_{spark} using a 4:1 RyR:LCC stoichiometry for 1 (top panel) or 4 (bottom panel) ICs, where the bar colours correspond to the AP waveform shown in A. In the presence of 1 IC, the mean latencies were 14.29 ± 0.12 ms (P_{spark} = 0.753) and 19.39 ± 0.16 ms (P_{spark} = 0.754) for HF and HF-long, respectively. In the presence of 4 ICs, the mean latencies were 8.40 ± 0.05 ms (P_{spark} = 0.998) and 10.19 ± 0.08 ms (P_{spark} = 0.995) for HF and HF-long, respectively. n_o were 2.33 ± 0.02 and

2.62 ± 0.03 for HF and HF-long, respectively. This means that P_{cpl} was reduced by ~20% (to 0.43 and 0.38 for HF and HF-long, respectively). 5,000 simulations were carried out for each AP waveform. C) I_{Ca} during the AP from 50,000 LCCs and proportion involved in triggering Ca^{2+} transients (shaded regions).

Supplementary References

[1] T. O'Hara, L. Virág, A. Varró, Y. Rudy, Simulation of the Undiseased Human Cardiac Ventricular Action Potential: Model Formulation and Experimental Validation, PLOS Comput. Biol. 7 (2011) e1002061. https://doi.org/10.1371/journal.pcbi.1002061.

[2] S.A. Grandy, S.E. Howlett, Cardiac excitation-contraction coupling is altered in myocytes from aged male mice but not in cells from aged female mice, Am. J. Physiol.-Heart Circ. Physiol. 291 (2006) H2362–H2370. https://doi.org/10.1152/ajpheart.00070.2006.

[3] I.R. Josephson, A. Guia, E.A. Sobie, W.J. Lederer, E.G. Lakatta, M.D. Stern, Physiologic gating properties of unitary cardiac L-type Ca^{2+} channels, Biochem. Biophys. Res. Commun. 396 (2010) 763–766. https://doi.org/10.1016/j.bbrc.2010.05.016.

[4] P. Asghari, D.R. Scriven, M. Ng, P. Panwar, K.C. Chou, F. van Petegem, E.D. Moore, Cardiac ryanodine receptor distribution is dynamic and changed by auxiliary proteins and post-translational modification, ELife. 9 (2020) e51602. https://doi.org/10.7554/eLife.51602.

[5] D.R. Laver, C.H.T. Kong, M.S. Imtiaz, M.B. Cannell, Termination of calcium-induced calcium release by induction decay: An emergent property of stochastic channel gating and molecular scale architecture, J. Mol. Cell. Cardiol. 54 (2013) 98–100. https://doi.org/10.1016/j.yjmcc.2012.10.009.

[6] M. Fill, D. Gillespie, Ryanodine Receptor Open Times Are Determined in the Closed State, Biophys. J. 115 (2018) 1160–1165. https://doi.org/10.1016/j.bpj.2018.08.025.
